# Prioritizing research for “One health - One world”

**DOI:** 10.1186/2049-9957-1-1

**Published:** 2012-10-25

**Authors:** Xiao-Nong Zhou

**Affiliations:** 1 National Institute of Parasitic Diseases, Chinese Center for Disease Control and Prevention, Shanghai, 200025, People’s Republic of China

## Abstract

Infectious diseases of poverty, a collective term coined for infections known to be particularly prevalent amongst poor populations, is increasingly used for neglected tropical diseases (NTDs) with special transmission routes, such as depending on vectors and/or intermediate hosts. The journal *Infectious Diseases of Poverty* (IDP) is launched to explore new avenues in research to better understand the relationship between infectious diseases and poverty, and to contribute to priority settings for plans to control them. Introducing the “One health - One world” concept, IDP will publish original and empirical work based on analyses of disease burdens, their distribution and research needs in this area. The new journal will not only bring out research articles but also scoping reviews and highlights of trans-disciplinary work undertaken to combat the infectious diseases of poverty, wherever in the world they exist.

## Multilingual abstracts

Please see Additional file 1 for translations of the abstract into the six official working languages of the United Nations.

## 

Ban Ki-Moon, Secretary-General of the United Nations noted in his address to the General Assembly on 21 September 2010, that “**the least developed countries represent the poorest and most vulnerable segment of humanity. They remain at the epicentre of the developmental emergency**.” Indeed, the current number of the least developed countries (LDCs) has doubled during the last 40 years and stands now at 48 condemning 1.3 billion people, more than a fifth of the world’s population, to currently live under the poverty line [1]. In the next 40 years, almost all population growth is projected to occur in the developing countries; they will, however, also bear the major share of the global burden of illness if nothing changes. For instance, life expectancy in the LDCs is still up to 30% shorter than it is in the industrialized part of the world and people do not generally have a healthy life [2]. To boot, life expectancy is declining rather than improving in some of the poorest countries. Indeed, there is a great risk that LDCs and their populations will continue to be impoverished and marginalized as they rarely enjoy the benefits brought by globalization, the information revolution and other technological advances [3]. This situation contributes strongly to the widening gap with respect to life expectancy between the LDCs and the industrialized world.

Climate change, globalization, urbanization, deforestation, and intensification of agriculture are all major drivers of environmental changes. They affect human health and create or widen gaps with regard to the socio-economic status between the rich and the poor in this world [3]. Indeed, the poor may not benefit from the ongoing economic development as much as others do. One of the most important consequences of this is the continued vulnerability of marginalized people to infectious diseases, which is fueled by factors such as poverty, low social status, environmental degradation and changing ecosystems [4]. For example, new evidence shows that the highest burden of zoonotic infectious diseases worldwide is mainly found in the poverty-stricken areas of the LDCs as well as low and low-middle income countries. For example, Ethiopia, Nigeria, Tanzania, and India have particularly high zoonotic disease burdens with widespread illness and death (Figure 1) [5]. The numbers of infected humans and animals cause huge economic losses through the establishment of vicious circles of disease, reduced work ability and poverty.

**Figure 1:**
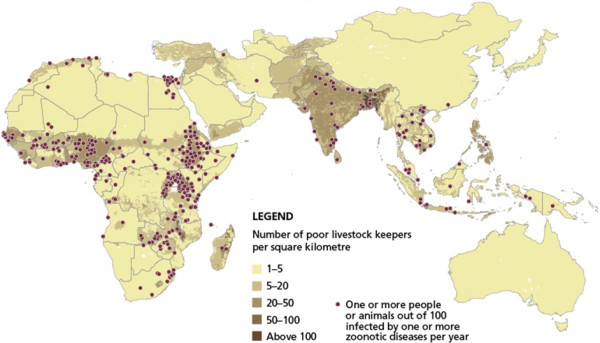
**The Greatest Burden of Zoonoses Falls on One Billion Poor Livestock Keepers. **An International Livestock Research Institute (ILRI) study has shown that zoonotic diseases are major obstacles in the pathway out of poverty for one billion poor livestock keepers. These diseases cause 2.3 billion cases of human illness and 1.7 million human deaths a year. In poor countries, they infect more than one in seventh of all livestock animals every year [5].

In order to understand the relationship between disease and poverty, the term ‘infectious diseases of poverty (IDPs)’ has been coined to describe a number of diseases known to be more prevalent amongst poor populations [6]. It includes the so-called neglected tropical diseases (NTDs) about which Dr Margaret Chan, Director General of World Health Organization (WHO) in her keynote address to the Prince Mahidol Award Conference on 1 February 2007, said: ***“They are not new and frightening diseases, they are ancient…”, “They maim, debilitate, blind, disfigure, and kill. They permanently diminish human potential, and do so in large populations…. ”***[7]. The overarching concept of IDPs draws attention to these ‘ancient’ or (re-)emerging diseases (Table 1) and makes us recognize the need to focus our attention on the poor and the vulnerable, who are powerless to understand and combat the diseases affecting them. Poor people in the developing world must concentrate on providing for their daily subsistence and have little time or energy for fighting what degrade their environment and endanger their health [4].

**Table 1:** List of the infectious diseases of poverty, with information of ‘ancient’ diseases and emerging or re-emerging infectious diseases of poverty indicating the scoops considered within this journal

***Type of infection***	***Infectious disease of poverty***	***‘Ancient’ diseases***	***Emerging or re-emerging diseases***
**Viral infections**			
	Rabies	*	*
	Dengue		*(in some regions)
**Bacterial infections**			
	Tuberculosis	*	
	Buruli ulcer	*	
	Leprosy	*	
	Meningitis	*	
**Parasitic infections**			
***.Vector-born diseases***	Malaria	*	
	Chagas disease	*	*( in some regions)
	Human African trypanosomiasis	*	
	Onchocerciasis	*	*( in some regions)
	Lymphatic filariasis	*	
	Leishmaniasis	*	
***.Snail-born diseases***	Schistosomiasis	*	*
	Opisthorchiasis	*	*
	Clonorchiasis	*	*
	Paragonimiasis	*	*
	Fascioliasis	*	*( in some regions)
	Fasciolopsis	*	*
***.Water-born diseases***	Cryptosporidiosis	*	*
	Giardiasis	*	*
	Toxoplasmosis	*	*
***.Soil-transmitted helminthiasis***	Hookworm	*	
	Trichuriasis	*	
	Ascariasis	*	
	Enterobiasis	*	
***.Zoonotic diseases***	Taeniasis/cysticercosis	*	
	Echinococcosis	*	
	Zoonotic schistosomiasis	*	

According to *the First WHO Report on Neglected Tropical Diseases: Working to Overcome the Global Impact of Neglected Tropical Diseases*[8], it is estimated that about 3 billion people are affected by the IPDs and millions more are at risk. Some of these ‘ancient’ diseases are associated with high fatality rates, and some result in long-term, chronic sequels such as cancer or other devastating changes affecting quality of life and people’s ability to provide for themselves and their families. Moreover, some emerging or re-emerging infectious diseases have the highest impact on the poor in remote or rural regions where it is difficult to implement a quick response to interfere with disease transmission cycles. Dengue or Chagas disease, for instance, are spreading worldwide, but the highest intensity of transmission is occurring in vulnerable populations with low incomes and substandard homes [9,10].

Mortality due to the IDPs is only a partial indicator of the burden of these diseases, the most important issue being the social consequences that follow making the poor unable to improve their environment or change their social status [11]. For example, diagnosis and treatment of many NTDs, e.g. leishmaniasis and echinococcosis are expensive outlays forcing families of patients to sell their assets and take loans to pay for the care they need. This leads to further impoverishment and reinforcement of the vicious cycle of disease and poverty [12]. How to integrate the infectious diseases control programmes into a universal health coverage system becomes a critical issue, when more than 150 million people worldwide currently suffer severe financial hardship each year due to illness and inability to pay for the medical services or medicines they need.

The principles and actions that can break this vicious cycle are known: (a) extended universal health coverage by public health providers, (b) sustainable development of a national programme that suits the LDCs’ own national conditions, facilitated by the international community and enforced by the LDCs themselves, (c) local political commitment with strong implementation of a reasonable strategy for the national programme through inter-sectoral action [13-15]. An international agreement on the need for all countries to commit themselves to achieving sustainable development has been made by the United Nations Conference on Sustainable Development (Rio + 20), which underlines the vital need for universal health coverage including policies to prevent, protect and promote a sustainable public health agenda [16]. We must ask ourselves, why are these principles not implemented?

Scientific research is the critical driver for a functioning, innovative, and sustainable health system and only research can end this vicious cycle. A multi-disciplinary approach, coupled with a holistic view of the public health issues raised by the IDPs, is called for, but health research remains fragmented, undervalued and marginalized with no priorities established [17]. Lessons from Haiti and Rwanda have shown that most health interventions in resource-poor settings not only performed inefficiently but also resulted in less cost-effective solutions [18]. The excess morbidity and mortality caused by the IDPs remain a visible indictment of the current fragmented approach, even though much discussion on how to break the vicious cycle between the infectious diseases and poverty has taken place at the national, regional and global levels [6,19,20]. There is, however, a flickering of light at the end of the tunnel as national programmes are now aiming to overcome the global impact of the NTDs by efforts to control and eliminate the infectious diseases following the WHO roadmap, launched in early 2012 [21].

In April 2012, the WHO Special Programme for Research and Training in Tropical Diseases (TDR) published the *Global Report for Research on Infectious Diseases of Poverty (*referred to as *Global Report* in the following text*)*, which advocates a paradigm shift in the approach to the control of the infectious diseases of poverty [6]. This report supports the view that multiple research perspectives, including health systems, environmental and social issues, agriculture, basic research and innovation, even research funding patterns and gaps, would allow the design of appropriate public health responses to the threat of infectious diseases affecting the poor [6]. It might seem obvious to advocate for a holistic, interdisciplinary approach including research; however, we have to deal with the historic legacy of the disease-by-disease, “silo” approach, adopted by control programmes and donors for so many years [22].

Developed over three years with the help of a “Think Tank” of 130 experts convened by TDR, the *Global Report* identified research-related actions that policy-makers, donors and researchers should focus on if the public health challenges of IDPs are to be met [6]. The report detailed the drivers of infectious diseases in poor populations and highlighted how advances in science and technology could be used to meet the challenges of controlling these diseases [22]. The authors of the *Global Report* recommended a “One Health - One World” approach to set the stage for a new strategic direction for research on infectious diseases pronouncing that “the need for intersectoral collaboration is now urgent. Funding priority should be given to research that adopts inter-disciplinary approaches; encourages collaboration between government ministries and agencies; and better incorporates ecology into disciplines – including public health, medicine, social sciences, veterinary sciences and agriculture.”

The journal ***Infectious Diseases of Poverty*** (IDP) ( http://www.idpjournal.com), for which I have the honor to serve as Editor-in-Chief, aims to build on the “One health - One world” approach to publish original and empirical work, including scoping reviews and original articles on trans-disciplinary research to combat the infectious diseases which affect mainly the poor populations. Our objectives are to:

• Review a wide range of topic areas, methods and strategies aiming at essential public health questions related to the IDPs;

• Identify and assess the research base underlying important current and future public health options, choices and decisions in relation to the IDPs;

• Identify and highlight information divergencies and research gaps that hinder progress towards new interventions for particular public health problems;

• Facilitate the much needed dialogue between policy makers, public health practitioners, control staff and academic researchers and their donors;

• Provide an advocacy platform for the translation of new knowledge into policy, propose research priority settings, and promote large-scale programmes to combat the IDPs.

The National Institute of Parasitic Diseases, China CDC, based in Shanghai, P.R. China [23], which has an internationally recognized track record of research on parasitic diseases and has been a ‘WHO Collaborating Centre on Malaria, Schistosomiasis and Filariasis’ for more than 30 years, has taken the lead in establishing this journal, which aims to complement the knowledge sharing and dissemination activities carried out by the TropIKA.net knowledge platform ( http://www.tropika.net) over the last four years. The IDP journal and TropIKA.net will be closely linked in their endeavors and contents. The IDP journal retains a large part of the experts that constituted the TDR Think Tank for the *Global Report* on its Editorial Board. The journal will promote work leading to research priorities in regions and countries through the commissioning of scoping reviews that can guide policy makers. In collaboration with WHO/TDR, it will strive to build capacity among young researchers to review existing research and identify knowledge gaps in the field of infectious diseases that affect their countries. IDP will also draw upon the large pool of expertise in the North and in the South to assist with the task and publish high-quality content.

IDP is an open-access, peer-reviewed journal published by BioMed Central. It is scheduled to be launched during the Second Global Symposium on Health Systems Research in later October 2012. For the first three years, the publication costs will be covered by the National Institute of Parasitic Diseases, Chinese Centre for Disease Control and Prevention.

IDP welcomes and actively seeks opportunities to work cooperatively with all groups of stakeholders engaged in research on infectious diseases, either scientific investigators, practicing physicians, decision-makers, research funders, academic society patient advocacy group or educational organization. Please join us - you are welcome!

## Supplementary Material

Additional file 1 Multilingual abstracts in the six official working languages of the United Nations. Click here for file
