# Supplementary material for: Prioritizing research for “One health - One world”
Source: Infect Dis Poverty. 2012 Oct 25;1:1. doi: 10.1186/2049-9957-1-1 (PMC3710101; doi:10.1186/2049-9957-1-1)
Supplement: Additional file 1 — Multilingual abstracts in the six official working languages of the United Nations. [file 2049-9957-1-1-S1.pdf]

Translation of the abstract into the six official working languages of the United Nations

أولوية البحث من أجل" صحة واحدة-عالم واحد"

كسياو-نونغ زو

ملخص

تعتبرالأمراض المعدية المرتبطة بالفقر مصطلحا جامعا صيغ ليعبر عن العدوى المعروفة والسائدة خاصة في المجتمعات الفقيرة، وهو يستعمل بشكل متزايد حول الأمراض الاستوائية المهملة(NTDP) مع طرق إنتقالها خاصة، كالإعتماد على النواقل و/أو الوسيط الناقل للمرض. تم إطلاق مجلة الأمراض المعدية المرتبطة بالفقر من اجل إستكشاف سبل جديدة عند البحث للوصول لفهم أكثر للعلاقة القائمة بين الأمراض المعدية والفقر والمساهمة في تحديد الأولويات عند وضع برنامج لمراقبتها. ستقوم مجلة الأمراض المعدية المرتبطة بالفقر بإدخالها مفهوم"صحة واحدة-عالم واحد" ، بنشر أعمال أصلية واختبارية قائمة على تحليل أعباء المرض و توزيعه وتحديد الحاجة للبحث في هذا الميدان. لن تقوم هذه المجلة فقط بإصدار مقالات بل ستتفحص المراجعات وتبرز العمل المتعدد التخصصات الذي آضطلع به من أجل محاربة الأمراض المعدية المرتبطة بالفقر أينما وجدت في العالم.

Translated from English version into Arabic by malika2012, through

TRANSLATORS
WITHOUT BORDERS

以“同一世界、同一健康”为目标确定研究优先领域

周晓农

摘要

“贫困所致传染病”是就主要流行于贫困人群感染性疾病的组合性术语，常用“被忽略热带病（NTDs）”来表示，其具有特殊的感染途径，如以媒介和/或中间宿主传播。贫困所致传染病杂志（IDP）的出版，旨在如何更好地研究传染病与贫困间的关系、为控制贫困所致传染病制定工作计划中优先领域探索新的研究方法。IDP 杂志将引入“同一世界、同一健康”之理念，发表与疾病负担分析与分布、研究需求等方面的原创性和经验性文章。本杂志不但发表研究类的文章，还将发表以控制全球现有贫困所致传染病的交错领域方面的勘域综述（scoping review）。

Translated from English version into Chinese by Zhou Xiao-Nong

« Un monde, une seule santé » : faire de la recherche une priorité

Xiao-Nong Zhou

Résumé

Le terme de maladies infectieuses liées à la pauvreté, qui regroupe les infections particulièrement prévalentes dans les populations pauvres, est de plus en plus utilisé pour les maladies tropicales négligées (MTN) avec voies de transmission spéciales, par exemple via des vecteurs et/ou des hôtes intermédiaires. Le journal *Infectious Diseases of Poverty* (**IDP**), ou maladies infectieuses liées à la pauvreté) a pour objectif d'explorer de nouvelles voies de recherche afin de mieux comprendre le lien entre les maladies infectieuses et la pauvreté, mais aussi de contribuer à la définition de priorités pour la mise en place de plans destinés à les contrôler. Sur la base du concept « Un monde, une seule santé », IDP publiera des travaux originaux et empiriques reposant sur des analyses du fardeau que représentent ces maladies, de leur répartition et des besoins en recherche dans ce domaine. Seront inclus non seulement des articles de recherche, mais aussi des analyses et des résumés de travaux transdisciplinaires réalisés pour combattre les maladies infectieuses liées à la pauvreté, dans tous les pays dans lesquels elles sont observées.

Translated from English version into French by ecarliez, through

TRANSLATORS
WITHOUT BORDERS

Приоритизация научных исследований в области концепции «Одно здоровье - Один мир»

Сяо-Нонг Чжоу

Резюме

«Инфекционные болезни нищеты», общий термин для инфекций, наиболее распространенных среди бедных стран, все больше используется в отношении «забытых» тропических болезней (NTDs), имеющих особые пути заражения, зависящие от носителей болезней и промежуточных носителей болезней. Целью издания журнала *«Инфекционные болезни нищеты»* (**IDP**) является изучение новых направлений в области научных исследований для лучшего понимания взаимосвязи между инфекционными болезнями и нищетой и определения приоритетов для программ по борьбе с ними. Представляя концепцию «Одно здоровье - Один мир», IDP будет публиковать оригинальные и эмпирические работы, основанные на анализе бремени болезней, их распределения и потребностей в проведении научных исследований в этой области. Новый журнал предоставит место не только научно-исследовательским статьям, но и публикациям предварительных обзоров и основных направлений междисциплинарной работы, направленной на борьбу с инфекционными болезнями нищеты, где бы в мире они ни существовали.

Translated from English version into Russian by OWatts, through

TRANSLATORS
WITHOUT BORDERS

Prioridad a la investigación para "Una salud - Un mundo"

Xiao-Nong Zhou

Resumen

Enfermedades infecciosas de la pobreza, un término colectivo que suele usarse para las infecciones que se sabe que son particularmente frecuentes entre las poblaciones pobres, se utiliza cada vez más para las enfermedades tropicales desatendidas (ETD) con vías de transmisión especiales, como las que dependen de vectores y / o huéspedes intermediarios. La publicación *Enfermedades Infecciosas de la Pobreza* (EIP) se lanza para explorar nuevos caminos en la investigación, para comprender mejor la relación entre las enfermedades infecciosas y la pobreza, y para contribuir a establecer la prioridad de los planes para su control. Al presentar el concepto "Una salud - Un mundo", EIP publicará trabajos originales y empíricos basados en el análisis de las cargas de las enfermedades, su distribución y las necesidades de investigación en este área. La nueva publicación no sólo pondrá en evidencia los artículos de investigación, sino también revisiones sintéticas y aspectos destacados de la labor transdisciplinaria emprendida para combatir las enfermedades infecciosas de la pobreza, en cualquier lugar del mundo en el que existen.

Translated from English version into Spanish by Susana Rosselli, through

TRANSLATORS
WITHOUT BORDERS
